# High-Density Linkage Map Construction and QTL Identification in a Diploid Blueberry Mapping Population

**DOI:** 10.3389/fpls.2021.692628

**Published:** 2021-06-21

**Authors:** Xinpeng Qi, Elizabeth L. Ogden, Hamed Bostan, Daniel J. Sargent, Judson Ward, Jessica Gilbert, Massimo Iorizzo, Lisa J. Rowland

**Affiliations:** ^1^Genetic Improvement of Fruits and Vegetables Laboratory, Beltsville Agricultural Research Center-West, United States Department of Agriculture, Agricultural Research Service, Beltsville, MD, United States; ^2^Department of Horticultural Science, Plants for Human Health Institute, North Carolina State University, Kannapolis, NC, United States; ^3^Driscoll’s Inc., Watsonville, CA, United States

**Keywords:** *Vaccinium*, genetic map, fruit quality, plant development, chilling requirement, cold hardiness

## Abstract

Genotyping by sequencing approaches have been widely applied in major crops and are now being used in horticultural crops like berries and fruit trees. As the original and largest producer of cultivated blueberry, the United States maintains the most diverse blueberry germplasm resources comprised of many species of different ploidy levels. We previously constructed an interspecific mapping population of diploid blueberry by crossing the parent F_1_#10 (*Vaccinium darrowii* Fla4B × diploid *V. corymbosum* W85–20) with the parent W85–23 (diploid *V. corymbosum*). Employing the Capture-Seq technology developed by RAPiD Genomics, with an emphasis on probes designed in predicted gene regions, 117 F_1_ progeny, the two parents, and two grandparents of this population were sequenced, yielding 131.7 Gbp clean sequenced reads. A total of 160,535 single nucleotide polymorphisms (SNPs), referenced to 4,522 blueberry genome sequence scaffolds, were identified and subjected to a parent-dependent sliding window approach to further genotype the population. Recombination breakpoints were determined and marker bins were deduced to construct a high density linkage map. Twelve blueberry linkage groups (LGs) consisting of 17,486 SNP markers were obtained, spanning a total genetic distance of 1,539.4 cM. Among 18 horticultural traits phenotyped in this population, quantitative trait loci (QTLs) that were significant over at least 2 years were identified for chilling requirement, cold hardiness, and fruit quality traits of color, scar size, and firmness. Interestingly, in 1 year, a QTL associated with timing of early bloom, full bloom, petal fall, and early green fruit was identified in the same region harboring the major QTL for chilling requirement. In summary, we report here the first high density bin map of a diploid blueberry mapping population and the identification of several horticulturally important QTLs.

## Introduction

Blueberry is a woody perennial shrub. Commercial types of blueberry are native to North America and belong to the *Cyanococcus* section of the genus *Vaccinium* in the heath family *Ericaceae*. The major commercial types in the U.S. are cultivars of *V. corymbosum* L. (tetraploid highbush blueberry) and *V. virgatum* Ait. (hexaploid rabbiteye blueberry; syn. *V. ashei* Reade), and wild, managed stands of *V. angustifolium* Ait. (tetraploid lowbush blueberry). Highbush blueberry cultivars can be further classified into northern and southern types, depending on their chilling requirements and, thus, the geographical areas where they can be grown. Southern highbush, with low chilling requirements, have been developed through the introgression of the low-chilling southern diploid species *V. darrowii* Camp into the tetraploid *V. corymbosum* background (Rowland et al., 2014).

Of the cultivated types, highbush blueberry is the most important commercially. Selective breeding of highbush blueberry began in the early 1900’s in New Jersey through the efforts of the USDA, in collaboration with blueberry growers (Paul and Childers, 1966). Even today developing a new blueberry cultivar through traditional breeding requires a decade or more of work from original cross to cultivar release (Gallardo et al., 2018), due to long generation times, high ploidy levels, and high genome heterozygosity. To speed up the breeding process, marker-assisted breeding has proven to be effective and efficient in some major agronomic crops (Wang et al., 2019). Recently, quantitative trait loci (QTLs) for development and fruit quality traits have been mapped in several berry crops, including cranberry (Georgi et al., 2013; Daverdin et al., 2017; Diaz-Garcia et al., 2018a,b), which is closely related to blueberry in the *Vaccinium* genus, raspberry (Paterson et al., 2013; Foster et al., 2019), blackberry (Castro et al., 2013), and strawberry (Lerceteau-Köhler et al., 2012).

For many years, our laboratory has been working on understanding the genetic control of important horticultural traits in blueberry breeding (Rowland et al., 1999). We have previously constructed a relatively low-density molecular marker-based genetic map of a diploid blueberry biparental population that segregates for chilling requirement, cold hardiness, and many fruit quality and plant development traits (Rowland et al., 2014, 2020). We constructed this pseudo-backcross interspecific mapping population by crossing a hybrid (F_1_#10) of a low-chilling, freezing-sensitive *V. darrowii* selection Fla4B and a high-chilling, freezing tolerant diploid *V. corymbosum* selection W85–20, to another diploid *V. corymbosum* selection W85–23. A true F_2_ or backcross population would be impossible to generate for mapping due to self-sterility (Galletta and Ballington, 1996) and inbreeding depression (Krebs and Hancock, 1988) in diploid blueberry. The first low-density map of this population, consisting of 265 molecular markers, primarily simple sequence repeats (SSRs) and expressed sequence tag-polymerase chain reaction (EST-PCR) markers, was useful for identifying a major QTL for chilling requirement and a major QTL for cold hardiness (Rowland et al., 2014). A later map of this same population, consisting of 409 markers, primarily SSRs from blueberry and cranberry, allowed for comparative genetic mapping between blueberry and cranberry and found an exceptionally high degree of synteny and collinearity between the genomes (Schlautman et al., 2018).

In recent years, single nucleotide polymorphism (SNP) markers have been used to generate a medium density genetic map of a tetraploid highbush blueberry breeding population (cross between “Draper” and “Jewel”) (McCallum et al., 2016) and a very high density genetic map of a tetraploid southern highbush blueberry population (derived from the cross “Sweetcrisp” × “Indigocrisp”) (Cappai et al., 2020). Significant QTLs have been identified for traits related to machine harvesting in the southern highbush population (Cappai et al., 2020), and genome-wide association studies (GWAS) have also recently been successfully used to identify QTLs in blueberry related to fruit quality and aroma (Ferrão et al., 2018, 2020).

In this current study, we employed a Capture-Seq technology, with an emphasis on probe design in predicted gene regions, to genotype our diploid blueberry mapping population. A total of 160,535 SNPs, referenced to the diploid blueberry draft genome (Bian et al., 2014), were called and a parent-dependent sliding window approach, similar to Huang et al. (2009), was applied to genotype the whole population. A high density blueberry linkage map was constructed comprised of 17,486 SNPs. Of 18 horticultural traits phenotyped in this population, QTLs that were significant over at least 2 years were identified for chilling requirement, cold hardiness, and fruit quality traits of color, scar size, and firmness. Genes in the vicinity of these QTLs were also identified, which will allow the testing of best candidates for these traits in future experiments.

## Materials and Methods

### Plant Material and Phenotypic Evaluation

Our diploid blueberry pseudo-backcross interspecific mapping population (Supplementary Figure 1), which segregates for mid-winter cold tolerance, chilling requirement, fruit quality, and plant development traits (Rowland et al., 2014, 2020) was used for constructing a SNP-based genetic linkage map and for QTL identification. The population was developed by crossing an interspecific F_1_ plant named F_1_#10 [*V. darrowii* selection Fla4B (low chilling, cold sensitive, evergreen) × diploid *V. corymbosum* selection W8–20 (high chilling, cold hardy, deciduous)] to another diploid *V. corymbosum* W85–23 (also high chilling, cold hardy, deciduous) (Rowland et al., 2014). The grandparent W85–20 was the plant used to develop the diploid blueberry draft genome (Bian et al., 2014). The mapping population was clonally propagated to give three to four clones of each individual on average. Approximately 120 genotypes from this cross (F_1_#10 × W85–23) are maintained in a greenhouse in 4 to 12 liter pots at the Beltsville Agricultural Research Center-West, Beltsville, MD. The population is not maintained in the field because many of the individuals are extremely cold sensitive and would not withstand the winter temperatures.

The mapping population, as well as parents and grandparents, were phenotyped over multiple years (2009–2019) for 18 traits at the USDA-ARS in Beltsville, MD. Traits included chilling requirement, cold hardiness, timing of various stages of development (flower bud, leaf bud, and fruit development), and various fruit quality traits (weight, diameter, color, scar size, firmness, flavor, and soluble solids). Phenotypic data collection, distribution, correlation, and heritability analyses were described in detail previously (Rowland et al., 2014, 2020). Briefly, all phenotypic evaluations used a completely randomized experimental design. For the cold hardiness evaluations, we moved plants from a heated hoop house to an unheated hoop house in early winter, cut shoots after cold exposure (500–550 h of chilling), and then subjected the detached shoots to a freeze-thaw regime, which included exposure to a range of freezing temperatures, to determine the temperature resulting in 50% injury of flower buds (LT50s). For the chilling requirement evaluations, we cut shoots from plants in the unheated hoop house after every 200 h of chilling (200–1,000 h) and then rated them for floral bud development after 3 weeks in a warm environment in order to determine the chill units required for 50% of the buds to break (CR50). Evaluations were done in 2009 and 2010 for cold hardiness, and 2011–2013 for chilling requirement (Rowland et al., 2014).

Evaluation of the population for the remaining 16 traits (2012–2019) was described in detail in Rowland et al. (2020). For the traits that required fruit development, a bumble bee hive was placed in the hoop house to ensure pollination. These traits included fruit quality traits that were scored based on rating scales from 1 to 9 of random samples of 10–12 berries for fruit color (black to bright, light blue color), scar (large, deep wet scar to small, dry scar), firmness (very soft to very firm fruit by gently squeezing; this trait is abbreviated Firm in tables and figures), and flavor (very tart to very sweet). Other objectively measured fruit quality traits included weight, diameter (Dia), soluble solids (SS), and firmness (20 S Firm stands for the force to compress the berry by 20% equatorially using a texture analyzer; 3 mmFirm stands for the force for a 1 mm probe to penetrate berries to 3 mm also using a texture analyzer). For these traits, 20–25 berry samples were used. Color, scar, and weight were evaluated over 6 years (2012, 2013, 2015, 2017–2019); Firm and flavor were evaluated over 4 years (2012, 2013, 2015, and 2017). Evaluations of Dia, SS, 20 S Firm, and 3 mm Firm were performed over 3 years (2017, 2018, and 2019).

The remaining seven traits included traits related to timing of leaf bud, flower bud, and fruit development. Stages were abbreviated in tables and figures as: shoot expansion (SE), early bloom (EB), full bloom (FB), petal fall (PF), early green fruit (EG), late green fruit (LG), and > 75% blue fruit (75 BLUE). These stages are defined on the MSU Extension Growth Stages Table^1^. Time from EB to FB (EB to FB), EB to 75 BLUE (EB to75 BLUE), and FB to 75 BLUE (FB to75 BLUE) was also calculated and used in the subsequent QTL analysis. Plants were evaluated on a weekly basis as vegetative and floral buds began to develop. The day of the year (Julian day) when > 50% of buds reached each stage was recorded for each genotype. These developmental/growth stage traits were evaluated in 2012 and 2013 (Rowland et al., 2020).

### Extraction of DNA and Performance of Capture-Seq

DNA was extracted from young leaf tissue of 117 mature individuals of the mapping population, along with the parents and grandparents as described previously (Rowland et al., 2003) using a modified CTAB procedure (Doyle and Doyle, 1987). DNA samples were shipped to RAPiD Genomics (Gainesville, FL, United States), where quantity and quality were assessed. Library preparation and Capture-Seq were then performed using PCR primers designed to amplify a defined set of regions within the blueberry genome. Approximately 80,000 unique blueberry 120-mer probe sequences were used, with a single probe sequence every 5 kb region of the blueberry (W85–20) reference genome (Bian et al., 2014).

### Read Filtering, Mapping and SNP Calling

Raw sequenced reads were trimmed using Trimmomatic (version 0.36) (Bolger et al., 2014). Reads containing any 15 bp window of an average sequencing quality score less than 15 were considered low quality and not used for further analysis. Clean reads were mapped to the blueberry genome using Burrows-Wheeler Aligner (BWA-mem) (Li and Durbin, 2009). The largest 7,162 scaffolds (top 95% of total length) of the publicly available diploid blueberry genome assembly (Bian et al., 2014) were used as a mapping reference.

After mapping, raw variants were called using Genome Analysis Toolkit (GATK HaplotypeCaller) (McKenna et al., 2010). SNPs were then obtained by filtering raw variants using Variant Call Format Tools (VCFtools) (Danecek et al., 2011). Only biallelic sites were maintained, with minimal mapping and genotype quality of 30 and mean aligning depth of 3–750. SNP sites with more than 50% missing data were also excluded. SNPs were then further filtered based on parental genotype criteria: (I) no SNPs could have missing data for parental genotypes; (II) at least one parental genotype must be heterozygous. Progeny genotypes were then checked to exclude SNP sites with distorted segregation using a Chi-square test (df = 1, chi-square value of 3.84 for marker type of “AA” × “AB” and “AB” × “AA,” df = 2, chi-square value of 5.99 for marker type of “AB” × “AB”).

### Parent-Dependent Sliding Window and Map Construction

Genotype block boundaries were first determined by a sliding window approach, similar to that described in Huang et al. (2009), applied to the two parents. Laddered window sizes from 5 to 19 continuous SNPs were evaluated, and a window size of 15 continuous SNPs was determined to be the best for maintaining heterozygosity of the two parents. A step size of one SNP was applied to slide each window along each reference scaffold. The window genotype was assigned as the dominant SNP, or assigned as “missing” if there was no dominant SNP. A genotype block was then determined by looping adjacent sites of the same window genotype.

Sequencing data for the whole population was then corrected according to each genotype block identified. For each individual, a bin was identified as an adjacent genomic region with the same genotypic profile generated by the sliding window approach. Recombination breakpoints were determined by inspection of the recombination pattern of the population. This parent-dependent sliding window analysis was performed using in-house scripts written in Perl.

Grouping of marker bins was performed using the R package OneMap version 2.1.3 (Margarido et al., 2007), with a recombination fraction maximum of 0.35 and logarithm of the odds (LOD) of 12. Genetic distances and order of marker bins for each linkage group were calculated by OneMap, using the Kosambi mapping function.

### Synteny Analysis

The tetraploid blueberry genome sequence (Colle et al., 2019) was downloaded and used for synteny analysis. Flanking sequences of 150 bp were retrieved from the diploid blueberry draft genome scaffolds (Bian et al., 2014). Syntenic hits between the diploid genetic map and the tetraploid blueberry genome were determined by BlastN (Zhang et al., 2000) (version 2.10.1+). The BlastN results where at least 100 bp out of 150 bp of the flanking sequence hit the tetraploid genome were considered valid hits. Syntenic hits were visualized by Circos (Krzywinski et al., 2009).

Synteny between the previous map of the same population based on SSR and EST-PCR markers (Schlautman et al., 2018) and this bin map was evaluated by map integration. The grouping and genetic distance calculations among bins, combined with the mapped molecular markers from the 2018 map, was conducted employing OneMap version 2.1.3 (Margarido et al., 2007) with the same parameters as described above. The shared markers between the integrated map and bin map were used as anchors for synteny blocks.

### QTL Identification and Retrieval of Gene Sequences in QTL Regions

Quantitative trait loci (QTLs) for each phenotyped trait were analyzed using QTLCartographer^2^ (version 1.14d). A scanning window length of 10 cM and a walking step of 0.5 cM were used. The QTLs were defined using a significance level of 0.05 determined by 1,000 permutations, and QTL boundaries were defined by the nearest bins adjacent to peak LOD scores using the standard LOD 1-drop rule (Li, 2011).

Gene sequences located in the QTL regions, which were significant over at least 2 years, were retrieved according to the publicly available diploid blueberry (W85–20) genomic sequence and gene prediction information (Bian et al., 2014; Gupta et al., 2015). Functional annotation for each gene was conducted using BlastX (Altschul et al., 1997) (version 2.10.1+) to the National Center for Biotechnology Information (NCBI) non-redundant protein sequences (nr) database.

## Results

### Population Sequencing, Read Mapping and SNP Calling

A diploid blueberry interspecific mapping population (Supplementary Figure 1), developed by crossing the parent F_1_#10 (*V. darrowii* Fla4B × *V. corymbosum* W85–20) with the parent W85–23 (*V. corymbosum*), was used to develop a high density genetic linkage map. Capture-Seq technology was used to sequence the two parents, two grandparents (Fla4B and W85–20), and 117 progenies of this population, yielding 131.7 Gbp clean reads after quality trimming. Reads from the grandparent genotype W85–20 were mapped back to its own genome assembly, which is the diploid blueberry reference genome, to evaluate the sequencing error rate, which was 0.13%. The whole population was then mapped onto the reference genome, resulting in an average mapping score of 39.46 (Supplementary Table 1). Data from progeny plant 300–21 was excluded from further analysis due to a high rate of missing data (78.1%).

A total of 160,535 high quality SNPs, after excluding highly distorted sites by Chi-square test, were obtained. These SNPs were located on 4,522 blueberry scaffolds, which resulted in a SNP density of 4.08 SNPs per 10 Kbp of the reference genome. Identified SNPs were evenly distributed along each blueberry chromosome, which is similar to the distribution pattern of annotated blueberry genes (Supplementary Figure 2). This SNP set contained 50,116 sites where parent W85–23 was heterozygous, 90,341 sites where parent F_1_#10 was heterozygous, and 20,078 sites where both parents were heterozygous. Parent F_1_#10 was expected to be heterozygous at more SNP sites than parent W85–23, because F_1_#10 is an interspecific hybrid derived from crossing a *V. darrowii* selection (Fla4B) to a diploid *V. corymbosum* selection (W85–20).

### Parent-Dependent Sliding Window Approach and High Density Map Construction

**FIGURE 1:**
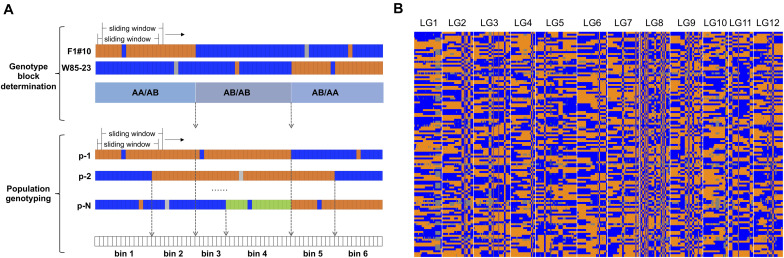
**(A)** Depiction parent-dependent sliding window approach. The length of the sliding window was 15 SNPs. **(B)** High density genetic map of the diploid blueberry F_1_ population. The orange color indicates the genotype”AA” at a locus, the blue color indicates the genotype “AB” at a locus, the green color indicates the genotype was different from parental genotypes, and the gray color indicates missing data.

The genomes of the two parents F_1_#10 and W85–23 were covered by the most sequencing data, of 49.7 and 30.6 million reads, respectively (Supplementary Table 1). After application of the sliding window approach to reduce sequencing noise, genotype blocks were identified (Figure 1A), resulting in a total of 1,038 blocks of “AA” × “AB” type, 684 blocks of “AB” × “AB” type, and 1,047 blocks of “AB” × “AA” type. From genotyping of the whole population, marker bins, containing markers with the same genotypic profile across the population, were determined within identified genotype blocks.

A high density diploid blueberry genetic map was constructed from the genotypic data (Figure 1B). The map was comprised of 17,486 SNP markers and 922 marker bins. The marker bins were grouped into 12 linkage groups (LGs), equal to the haploid chromosome number for diploid blueberry (Table 1 and Supplementary Table 2). The average number of SNPs per LG was 1457.2 in 76.8 marker bins. The total genetic distance of this map was 1,539.4 cM. The average genetic length of the 12 LGs was 128.3 cM, with the largest LG of 175.5 cM and the smallest LG of 101.9 cM (Table 1). The largest LG had a total of 2,377 SNPs in 119 marker bins (average of 20 SNPs per bin), while the smallest LG had a total of 1,466 SNPs in 77 marker bins (average of 19 SNPs per bin). A total of 25 gaps in the map were larger than 10 cM. These were most frequently found in the centromeric regions. The gaps were fairly evenly distributed across the LGs (Table 1).

**TABLE 1:** Summary statistics of high density diploid blueberry genetic map.

LG	Marker bins	SNP markers	Genetic length-cM (cM)	Gaps > 10 cM ccM cM	Synteny with tetraploid genome (VaccDscaff ID)	Synteny with 2018 map (LG)
1	91	1,680	116.74	1	1, 5, 8, 10	2
2	62	1,287	131.21	3	2, 3, 14, 25	6
3	85	1,764	156.26	3	4, 9, 35, 36	1
4	77	1,466	101.91	1	6, 37, 38, 39	4
5	119	2,377	175.47	3	7, 16, 18, 31	9
6	85	1,779	127.48	2	11, 15, 19, 24	5
7	87	1,452	134.52	2	12, 23, 40, 41	3
8	60	521	126.19	2	13, 30, 32, 42	8
9	72	1,247	137.41	2	17, 27, 34, 45	10
10	50	1,006	105.13	2	19, 20, 28, 44, 48	11
11	27	344	102.92	3	21, 26, 29, 33	12, 13
12	107	2,563	124.14	1	22, 43, 46, 47	7

*For each LG, this includes number of marker bins, number of SNP markers, genetic length in cM, number of gaps > 10 cM, synteny with tetraploid genome chromosomes, and synteny with LGs of previous genetic map (Schlautman et al., 2018).*

### Synteny Analysis

**FIGURE 2:**
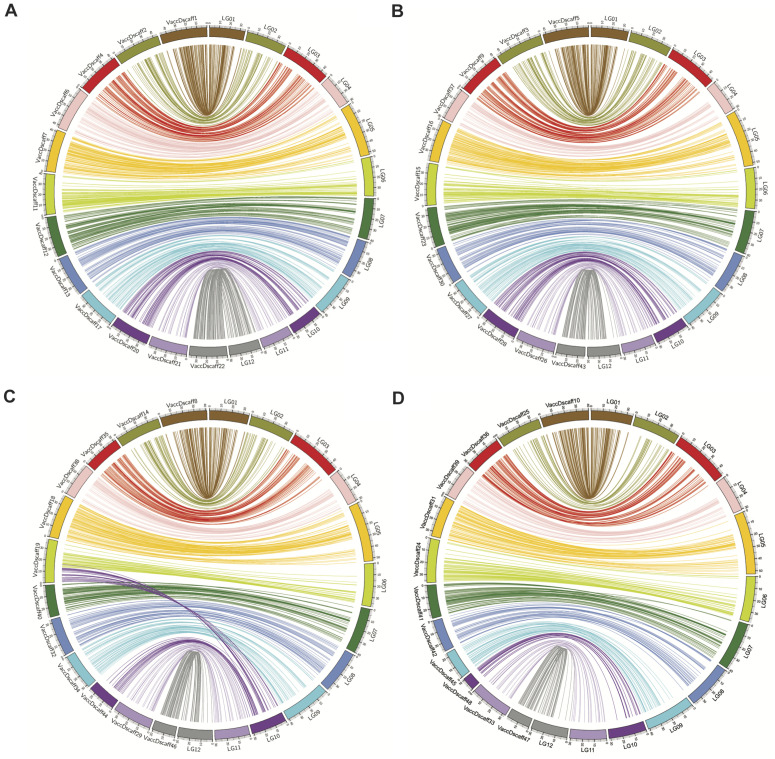
Syntenic hits between high density diploid blueberry genetic map and published tetraploid blueberry genome. **(A–D)** Show syntenic hits of diploid blueberry map LGs to the tetraploid blueberry genome haplotype subsets, 1–4, using Circos. VaccDscaff IDs refer to the different Vaccinium “Draper” genome scaffolds. Synteny of VaccDscaff IDs with the LG numbers are given in Table 1.

From the synteny analysis, linear syntenic hits were found for all 12 LGs of the diploid genetic map to the 4 × 12 chromosome sets of the tetraploid blueberry genome (Colle et al., 2019; Figure 2 and Supplementary Table 2). A translocation event in super scaffold 19 of the tetraploid genome was identified by syntenic hits with it to LG 06 and LG 10 of the diploid map (Figure 2C).

To check the map consistency with the previously published map of this same population, the 409 SSR and EST-PCR markers from the previously published genetic map (Schlautman et al., 2018) were combined together with the marker bins of the high density map, and an integrated map was constructed using the same parameters as those used for construction of the high density map. A total of 366 out of the 409 molecular markers of the 2018 map were resolved into 12 LGs together with the new marker bins. The two small LGs, LG 12 and LG 13 of the older map, grouped together to the same LG in the integrated map (Supplementary Figure 3). The 2018 map and the new high density map appeared collinear, with just a few exceptions, after integration.

### QTL Identified for Important Horticultural Traits

**FIGURE 3:**
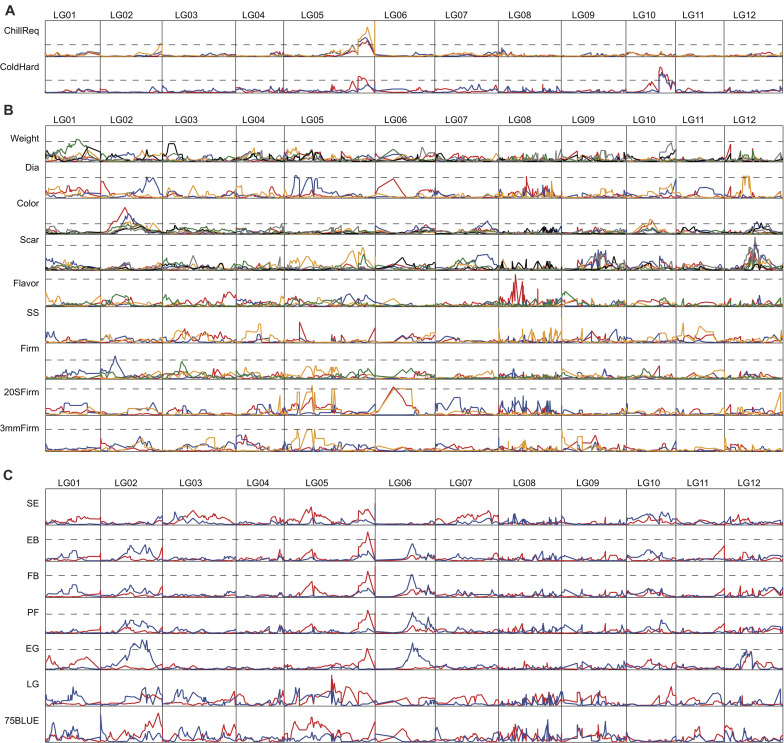
LOD score distribution of QTL along the 12 LGs of the high density genetic map. LOD cutoffs for traits with significant QTL are shown by the dashed lines. For traits with no significant QTLs, LOD cutoffs are not drawn. Abbreviations for traits are: chilling requirement, ChillReq; cold hardiness, ColdHard; diameter, Dia; soluble solids, SS; firmness rated by squeezing the berries, Firm; firmness measured with a Texture Analyzer by pressing the berries equatorially, 20 S Firm; firmness measured with a Texture Analyzer by puncturing the skin, 3 mm Firm; shoot expansion, SE; early bloom, EB; full bloom, FB; petal fall, PF; early green, EG; late green, LG; 75% blue, 75BLUE. LOD cutoff values for traits are: ChillReq, 7.65; ColdHard, 7.38; Weight, 11.52; Dia, 9.45; Color, 10.14; Scar, 11.89; Flavor, 10.52; SS, 20.98; Firm, 11.33; 20 S Firm, 16.50; 3 mm Firm, 12.21; SE, 16.20; EB, 11.16; FB, 15.98; PF, 12.89; EG, 11.25; LG, 8.81; 75 BLUE, 12.32. **(A)** QTL analysis of chilling requirement and cold hardiness. **(B)** QTL analysis of fruit quality traits. **(C)** QTL analysis of timing of developmental traits. Phenotypic data collected in different years is represented by different colored lines.

A QTL analysis was carried out using the high density map and phenotypic data collected over multiple years for 18 horticultural traits. Detailed results of all significant QTLs are presented in Figure 3 and Supplementary Table 3. A summary of QTLs supported by at least 2 years’ phenotypic data is shown in Table 2. The summary includes LOD cutoff and peak LOD scores, position of QTL, heritability of the trait, and percent of phenotypic variance and genotypic variance explained by the QTL. Considering all traits, a total of six QTLs were identified that were consistent over at least 2 years. These included one QTL for chilling requirement, one QTL for cold hardiness, two QTLs for color, one QTL for scar size, and one QTL for 20 S Firm. These six QTLs spanned five LGs (Table 2).

**TABLE 2:** Summary of QTLs supported by at least 2 years’ phenotypic data.

Trait ID	Cutoff LOD score	Peak LOD score	LG/QTL interval	H^2^	R^2^	R^2^/H^2^
ChillReq_2011	13.64	25.95	5/Bin113–119	0.86	0.3	0.35
ChillReq_2012	12.99	37.26	5/Bin113–119		0.3	0.35
ChillReq_2013	9.00	49.22	5/Bin113–119		0.4	0.47
ColdHard_2009	9.59	21.85	10/Bin32–36	0.88	0.27	0.31
ColdHard_2010	11.22	17.31	10/Bin32–36		0.22	0.25
Color_2012	13.08	30.50	2/Bin20–32	0.80	0.38	0.48
Color_2013	8.46	22.84	2,10/Bin20–32, Bin18–25		0.25/0.18	0.31/0.23
Color_2015	7.68	16.80	2,10/Bin20–32, Bin18–25		0.21/0.39	0.26/0.49
Color_2017	9.78	11.64	2/Bin20–32		0.13	0.16
Scar_2018	6.18	9.21	12/Bin79–80	0.67	0.11	0.16
Scar_2019	11.40	19.00	12/Bin79–80		0.44	0.66
20SFirm_2017	6.64	16.06	6/Bin4–10	0.47	0.42	0.89
20SFirm_2019	15.42	17.04	6/Bin4–10		0.63	1.00

*For each QTL, this includes Trait ID, cutoff LOD and peak LOD scores, LG on which the QTL is located, QTL interval, heritability (H^2^) of the trait, percent of phenotypic variance explained by the QTL (R^2^), and percent of genotypic variance explained by the QTL (calculated as R^2^/H^2^).*

One major QTL for chilling requirement and one major QTL for cold hardiness were detected on LG 05 and LG 10, respectively, and were consistent over all years evaluated, which included 3 years for chilling requirement (2011–2013) and 2 years for cold hardiness (2009–2010) (Figure 3A and Table 2). The percent of phenotypic variance explained by the QTL for chilling requirement (on LG05, interval of Bin113–119, average peak LOD of 37.5) averaged 33.3% over the 3 years evaluated. The percent of genotypic variance explained by the QTL for chilling requirement, calculated by dividing the percent of phenotypic variance explained by the heritability of the trait, averaged 39.0%. The percent of phenotypic variance explained by the QTL for cold hardiness (on LG10, interval of Bin32–36, average peak LOD of 19.6) averaged 24.5% over the 2 years evaluated. The percent of genotypic variance explained by the cold hardiness QTL was 28.0%. Another QTL for cold hardiness was detected in only 1 year (2009), and it overlapped with the chilling requirement QTL on LG05 (Figure 3 and Supplementary Table 3). This second cold hardiness QTL explained 19% of the phenotypic variance and 21.6% of the genotypic variance.

Several QTLs that were consistent over at least 2 years were also identified for the three fruit quality traits of color (LG02 and LG10), scar (LG12), and 20 S Firm (LG06) (Figure 3B and Table 2). The color QTL on LG02 (interval of Bin20–32, average peak LOD of 20.4) was consistent over 4 years of evaluation (from 2012 to 2017). It explained an average of 24.3% of the phenotypic variance and 30.4% of the genotypic variance. The color QTL on LG10 (interval of Bin18–25, average peak LOD of 19.8) was consistent over 2 years (2013 and 2015) and explained an average of 28.5% of the phenotypic variance and 35.6% of the genotypic variance. The QTL for scar, located on LG12 (interval of Bin 79–80, average peak LOD of 14.1), was consistent over 2 years (2018 and 2019) and explained on average 27.5 and 41.0% of the phenotypic and genotypic variation, respectively. Firmness was evaluated in our population using three different methods (see “Materials and Methods” section). A major QTL for firmness was identified using the 20 S Firm method, which measured the force to compress the fruit equatorially, that was consistent over 2 years (2017 and 2019). It was located on LG06 (interval of Bin4–10, average peak LOD of 16.6) and explained an average of 52.5% of the phenotypic variance and 94.5% of the genotypic variance. A common QTL for 20 S Firm and 3 mm Firm was also detected in 1 year on LG05 that explained about 35 and 44% of the phenotypic variance, respectively (Figure 3B and Supplementary Table 3). Regarding five of the other six fruit quality traits (Firm, Flavor, Weight, Dia, 3 mm Firm), significant QTLs were detected, but none of the significant QTLs were detected over multiple years (Figure 3B and Supplementary Table 3). Furthermore, no significant QTLs were detected for SS in any year.

We also attempted to identify QTLs for the seven developmental stages evaluated, from shoot expansion (SE) to 75% ripe fruit (75 BLUE), and for time from EB to FB, EB to75 BLUE, and FB to75 BLUE (Figure 3C and Supplementary Table 3). Even though no QTLs were identified that were consistent over a minimum of 2 years, it is noteworthy that a common QTL for EB, FB, PF, and EG, was identified in 2012 on LG05 in the same region harboring the QTL for chilling requirement (Figure 3C). Another common QTL for FB, PF, and EG was located on LG06 in a single year (2013) (Figure 3C). Although these QTLs were supported by only 1 year of phenotypic data, they were detected across multiple timing traits, and the one on LG05 overlapped with the major chilling requirement QTL and the minor cold hardiness QTL.

### Candidate Gene Search

The genes located in the vicinity of QTLs that were consistent over two or more years were retrieved according to the diploid reference genome sequence (Bian et al., 2014) and gene prediction information (Gupta et al., 2015). These included all QTLs listed in Table 2, for chilling requirement, cold hardiness, color, scar, and 20 S Firm. A total of 277 candidate genes located in the chilling requirement (LG05) QTL region (Supplementary Table 4), 112 candidate genes located in the cold hardiness (LG10) QTL region (Supplementary Table 4), 247 candidate genes located in the color (LG02) QTL region (Supplementary Table 5), 188 candidate genes located in the color (LG10) QTL region (Supplementary Table 6), 129 genes located in the scar (LG12) QTL region (Supplementary Table 7), and 214 candidate genes located in the 20 S Firm (LG06) QTL region (Supplementary Table 8) were captured and annotated. These lists of genes were scanned to identify the best candidate genes for each of the traits. Details of this follow in “Discussion” section.

## Discussion

Blueberry is a rapidly growing fruit crop and is now considered the second most important soft fruit crop after strawberry. Production currently exceeds 525,000 metric tons worldwide (Brazelton et al., 2017). To meet the demand for blueberries, the development of new genetic and genomic resources for use in marker-assisted selection and faster, higher throughput phenotyping for more efficient breeding of blueberry are needed. Genomic resources include biparental mapping populations and association panels for mapping QTLs for important traits to the blueberry industry. We previously developed a biparental diploid mapping population aimed at mapping cold hardiness and chilling requirement (Rowland et al., 2014). Most recently, the population has been phenotyped for several fruit quality traits (weight, diameter, color, scar, firmness, flavor, and soluble solids) and for timing of various stages of flower bud, leaf bud, and fruit development (Rowland et al., 2020). Most of these traits segregated and were distributed normally in the population, and most had moderate to high heritability. We, therefore, concluded that the population should be useful for identifying QTLs for many of these traits (Rowland et al., 2020).

In order to map QTLs in our diploid blueberry population, here we have constructed a high density genetic linkage map comprised of 17,486 SNPs, in 922 marker bins, and 12 LGs. The total genetic distance of the map is 1,539.4 cM, comparable to previously published genetic maps of blueberry (Rowland et al., 2014; McCallum et al., 2016; Schlautman et al., 2018). This map is comprised of more markers than previous SNP- and SSR-based maps of the tetraploid highbush blueberry “Draper” × “Jewel” population (McCallum et al., 2016). In this tetraploid population, the “Draper” map was comprised of 785 markers and the “Jewel” map was comprised of 536 markers. Very recently, a high density genetic linkage map comprised of 11,292 SNP markers was reported for a tetraploid southern highbush blueberry population “Sweetcrisp” × “Indigocrisp” (Cappai et al., 2020). The density of this map is comparable to our latest map described herein of the diploid population.

The synteny of our genetic linkage map with the four chromosome sets of the recently published tetraploid blueberry genome (Colle et al., 2019) was evaluated and is highly collinear. Interestingly, the synteny analysis also suggests a translocation event in super scaffold 19 of haplotype 3 of the tetraploid genome may have occurred, because of it having hits to two LGs of the diploid map, LG06 and LG10. This translocation could have happened during the blueberry genome evolution, perhaps during duplication of the whole genome (Wang et al., 2020), or it could be specific to one or a few tetraploid blueberry genomes. It could be specific to the “Draper” genome that was sequenced. To get some idea of how widespread this translocation is, we examined the synteny of the recently published high density tetraploid map (Cappai et al., 2020) to the tetraploid blueberry genome (Colle et al., 2019). By extracting 150 bp flanking sequences to the SNPs, which were provided in the Supplementary Table 1 from the tetraploid map paper (Cappai et al., 2020), and blasting them to the tetraploid blueberry genome, haplotype 3, the translocation was detected (Supplementary Figure 4). As can be seen in the figure, the tetraploid map LG10 had syntenic hits to regions of the VaccDScaff19 and the VaccDscaff44, just as LG10 of the diploid map did. This suggests the translocation was not present in the tetraploid mapping population and may be unique to the “Draper” genome.

After constructing the high density diploid map, we attempted to re-map QTLs for chilling requirement and cold hardiness and map, for the first time, QTLs for several plant development and fruit quality traits using the phenotypic data for the diploid population. The major QTLs for chilling requirement and cold hardiness detected previously using the 2014 map (Rowland et al., 2014) were confirmed on the new high density map, and they were consistent over all years of evaluation. The percentages of phenotypic variance explained by the major QTL for chilling requirement (on LG05) and major QTL for cold hardiness (on LG10) were also comparable to that seen previously, 17.7% for chilling requirement and 26.5% for cold hardiness (Rowland et al., 2014) vs. 33.3% for chilling requirement and 24.5% for cold hardiness herein.

After retrieving the gene sequences (from the diploid genome) in the vicinity of the chilling requirement and cold hardiness QTLs, we scanned through the lists of annotated genes. We did not find any genes that would be obvious candidates for controlling these traits such as, in the case of chilling requirement, flowering-related genes described in herbaceous plants like *CONSTANS 1*, *FLOWERING LOCUS C*, *FLOWERING LOCUS T*, *SUPPRESSOR of Overexpression of Constans 1*, *LEAFY*, *APETALA1*, and *FRUITFULL* (Fornara et al., 2010; Walworth et al., 2016). In the case of the cold hardiness QTL, we did not find major regulatory genes involved in the cold-response pathway of plants, like *CBF* or *ICE* transcription factors (Wisniewski et al., 2014). This may be because most of the genes from the draft diploid genome assembly are annotated as “hypothetical proteins”, probably because of the fragmented nature of the assembly. We did identify some candidate genes for future testing, however, such as genes induced during cold acclimation of blueberry in the vicinity of the cold hardiness QTL that we have reported previously, like genes encoding a zinc finger CCCH domain-containing protein, an ADP-ribosylation factor, and an ethylene-responsive transcription factor (Dhanaraj et al., 2007). We should be able to identify even more candidates from the forthcoming chromosome-level assembly of the diploid genome (Iorizzo et al., 2018), as the plant used for the diploid genome sequence is a grandparent of our diploid mapping population, W85–20.

In peach and related species, a group of *DAM* (*dormancy-associated Short Vegetative Phase (SVP)*-like *MADS*-box) genes have been shown to be responsible for a QTL for chilling requirement (Zhu et al., 2020). This QTL coincides with the *EVG* (*evergrowing* mutation) locus. We searched the diploid and tetraploid blueberry genomes for *DAM*-related genes and did not get any valid hits using BlastN. A search to the tetraploid genome predicted protein sequences using BlastP revealed three genes encoding proteins with fairly low similarity (identity of 45–51%) to DAM proteins, two on chromosomes with synteny to LG08 and one on a chromosome with synteny to LG05, but far away from the chilling requirement QTL. This suggests that *DAM*-related genes are probably not underlying the chilling requirement QTL in our mapping population.

In our previous study, where we mapped chilling requirement and cold hardiness using our low-density map, we found that the closest markers to the cold hardiness QTL were EST-PCR markers, leaf-00248 and berc 230. The 454 ESTs from which these markers were derived encode proteins with high similarity to a peptidy-prolyl *cis-trans* isomerase-like protein or cyclophilin and to a hypothetical protein with similarity to alpha/beta hydrolase superfamily, respectively (Rowland et al., 2014). Here, we searched the diploid blueberry genome and found that a gene with high similarity to berc 230 is located in the region of the cold hardiness QTL on LG10. Interestingly, allelic variants (W14/15) of a gene encoding an esterase, belonging to the alpha/beta hydrolase fold superfamily, has been shown to be associated with overwintering survival in perennial gentian plants (*Gentiana* L.), and the protein is highly expressed in dormant, freezing-tolerant overwintering buds (Hikage et al., 2016). Thus, the gene we found, with high homology to the EST berc 230, could be a good candidate for being responsible for the cold hardiness QTL and should be tested further.

Using our high density map, major QTL were also detected for fruit quality traits of color, scar size, and firmness (20 S Firm) that were consistent over multiple years. Regarding color, light blue-colored fruit is preferred over black-colored fruit in the blueberry industry and is due to the presence of a waxy coating or “bloom” on the berries. Thus, the color QTLs we identified are most likely associated with presence of the waxy coating. In another northern-adapted rabbiteye blueberry breeding population segregating for the waxy coating/light blue color, we found that presence of the waxy coating on the fruit was associated with high expression of the *FatB* gene, which encodes acyl-[acyl-carrier-protein] hydrolase, also known as palmitoyl-acyl carrier protein thioesterase. This gene is important for supplying fatty acids for very-long-chain fatty acid (VLCFA) biosynthesis, which, in turn, is required for wax biosynthesis (Qi et al., 2019). We searched for this gene in the diploid blueberry reference genome. The two scaffolds it is located on were not captured by our map; therefore, we could not determine if the gene is located in the region of the color QTL from the diploid draft genome alone. Therefore, we next searched for the *FatB* gene in the tetraploid genome. Interestingly, this resulted in four hits (maker-VaccDscaff2-snap-gene-247.42-mRNA-1, maker-VaccDscaff3-snap-gene-135.35-mRNA-1, maker-VaccDscaff14-snap-gene-209–38-mRNA-1, and maker-VaccDscaff25-augustus-gene-246.31-mRNA-1). Each of the four hits had high similarity identity (>90%), each were located on a different chromosome, and the four chromosomes were those that had synteny to LG02 of our map. The longest hit block of 893 bp was to VaccDscaff3. This *FatB* gene is in the region of the color QTL on LG02. The color QTL synteny block position in the tetraploid genome is VaccDscaff3: 11,201,628–17,643,422. The gene “maker”-VaccDscaff3-snap-gene-135.35-mRNA-1 is at VaccDscaff3: 13,498,894-13,524,611. Therefore, this gene is an excellent candidate for being responsible for the color QTL. Future work will involve comparing the sequence and expression of this gene in the plants of the mapping population with extreme opposite color phenotypes to determine its role in expression of fruit color.

It is still possible that other genes could be responsible for segregation of color in the population. We, therefore, scanned the diploid and tetraploid genomes for other genes in the vicinity of the color QTLs, which could be involved in wax biosynthesis or transport. Two genes, annotated as fatty acid hydroxylase and palmitoyltransferase from the tetraploid genome, in the vicinity of the color QTL on LG02, were identified as potential candidates. CER1 is a fatty acid hydroxylase and plays an important role in wax biosynthesis. A wax-less mutant of cabbage has recently been shown to have low levels of CER1-transcripts (Dong et al., 2019). However, the fatty acid hydroxylase we identified does not appear to have high similarity to CER1 from cabbage or Arabidopsis. Regarding the palmitoyltransferase, overexpression of a rice gene, *OsLCB2a1*, which encodes a subunit of a serine palmitoyltransferase, an enzyme important for sphingolipid biosynthesis, has been shown to result in higher wax deposition on leaves of transgenic Arabidopsis plants (Begum et al., 2016). From our scans of the diploid genome, a gene (CUFF.9282.1) in the color QTL region on LG02 was found, which was annotated as very-long-chain 3-ketoacyl-CoA synthase (KCS) on the Genome Database for Vaccinium (GDV) website^3^. This gene is also an excellent candidate for being responsible for the color QTL, as it encodes a key enzyme involved in VLCFA and cuticular wax biosynthesis (Beaudoin et al., 2009). These genes will all be tested further by comparing their expression in plants of the mapping population with extreme opposite color phenotypes.

Berry scar size was also phenotyped in the diploid population (Rowland et al., 2020). The scar refers to the opening left on the back of the berry where the pedicel detaches. The scar can be a site of pathogen entry, thus a small scar is desirable. A QTL for scar was located on LG12 that was consistent over 2 years of evaluation. Scanning through the list of annotated genes from the diploid genome in the scar QTL region, we identified several genes which encode proteins involved in lignin biosynthesis. These include caffeic acid 3-O-methyltransferase, cinnamate 4-hydroxylase, and UDP-glycosyltransferase. Interestingly, berry drop in table grape (*Vitis vinifera* L.) has been related to hardening and thickening of the pedicel by its lignification (Garcia-Rojas et al., 2018). Thus, these genes could be good candidates for underlying the scar QTL and should be tested further.

Fruit firmness was also evaluated in our population (Rowland et al., 2020), and a major QTL for firmness (using the 20 S Firm method) was identified on LG06 that was consistent over 2 years. By scanning through the list of annotated genes from the diploid genome in the 20 S Firm QTL region, we identified several genes encoding alpha-glucosidases and one encoding pectin methylesterase and pectin methylesterase inhibitor. These genes are related to fruit softening, and, interestingly, an alpha-d-glucosidase and pectin methylesterase have recently been shown to increase in wine grapes during berry softening triggered by an alternating high and low temperature regime (Coletta et al., 2019). Thus, these genes could be good candidates for underlying the 20S Firm QTL and should be tested further.

The timing of various stages of flower bud, leaf bud, and fruit development were also mapped in the diploid population using the high density map. A common QTL for timing of early bloom, full bloom, petal fall, and early green fruit was detected in 1 year, 2012, and it coincided with the major QTL for chilling requirement on LG05. Since this timing-related QTL was only significant in 1 year, however, its detection may depend on weather conditions and how conditions contributed to fulfillment of the chilling requirement, such as a particularly early or late satisfaction of the chilling requirement in 1 year vs. another. Many of these timing related traits were previously found to be correlated with each other, and timing of shoot expansion, early bloom, and full bloom were also found to be correlated with chilling requirement (Rowland et al., 2020).

In relation to other tetraploid blueberry mapping studies, a “Draper” × “Jewel” mapping population has been phenotyped for many of the same traits as the diploid blueberry population (Hancock et al., 2018), but has not yet been used to map QTLs, because the population size was thought to be too small. Efforts are underway, however, to increase its population size. Significant QTLs for traits related to machine harvesting have been recently identified in a southern highbush blueberry mapping population, “Sweetcrisp” × “Indigocrisp” (Cappai et al., 2020). Firmness-related QTLs were found on LG07 and LG08, and, thus, they did not overlap with the 20S Firm QTL on LG06 identified in our study. In addition, a Ripe Fruit Detachment Force (Blue FDF) QTL was found on LG12 in the tetraploid mapping study, but not in the same region as the scar QTL on LG12 of our study. Panels of southern highbush blueberry genotypes have also been used in genome-wide-association studies (GWAS) to identify candidate genes for fruit quality, aroma, and other traits (Ferrão et al., 2018, 2020). Fifteen SNPs have been identified that are associated with fruit size, scar diameter, soluble solids, pH, and flower bud density (Ferrão et al., 2018). A comparison of the genes associated with these SNPs to our results found that one of the genes associated with fruit size (gene.g14573.t1, hypersensitive-induced response protein 1-SPFH/Band 7/PHB domain) is located in the vicinity of a minor QTL for 3 mm Firm from our study. Interestingly, we previously found that 20S Firm was moderately correlated with fruit size (Weight and Dia) in our diploid mapping population (Rowland et al., 2020). In addition, two of the genes associated with flower bud density (CUFF13871.1 and 54064_g.1) in Ferrão et al. (2018) are located in the region of a minor QTL for Dia from our results.

Our future studies will involve attempting to increase QTL resolution and scanning genes in the vicinity of more QTLs using both the chromosome-level tetraploid and forthcoming diploid genome assemblies. Candidate genes for traits will be identified, and real-time qPCR performed to compare expression of these genes in progeny with contrasting phenotypes. Sequences of the best candidate genes will be compared as well in plants with extreme opposite phenotypes. In addition, as more of the same or similar traits are mapped in more blueberry populations, the QTLs and associated genes will be compared among populations to determine if the same genes are involved.

## Data Availability Statement

The datasets presented in this study can be found in online repositories. The names of the repository/repositories and accession number(s) can be found below: https://www.ncbi.nlm.nih.gov/, SRR11946900-SRR11947020, and https://www.ebi.ac.uk/eva/, PRJEB40575.

## Author Contributions

LR conceived and directed the study. LR, XQ, and MI designed the study. XQ analyzed sequences, called SNPs, filtered data, constructed the genetic linkage map, performed synteny analyses, mapped QTLs, and identified and annotated genes in the vicinity of QTLs. EO maintained the mapping population, collected leaf tissue, extracted DNA, and phenotyped the population. LR, MI, DS, HB, JW, and JG advised on various aspects of data filtering and analysis, map construction, and synteny analyses. All authors contributed to the writing of the manuscript and read and approved the manuscript.

## Conflict of Interest

DS, JW, and JG were employed by the company Driscoll’s Inc. The remaining authors declare that the research was conducted in the absence of any commercial or financial relationships that could be construed as a potential conflict of interest.

## Footnotes

1 https://www.Canr.msu.edu/blueberries/growing_blueberries/growthstages

2 https://brcwebportal.cos.ncsu.edu/qtlcart/

3 www.Vaccinium.org
