# Supplementary material for: High-Density Linkage Map Construction and QTL Identification in a Diploid Blueberry Mapping Population
Source: Front Plant Sci. 2021 Jun 21;12:692628. doi: 10.3389/fpls.2021.692628 (PMC8256855; doi:10.3389/fpls.2021.692628)
Supplement: Supplementary file 1 [file Data_Sheet_1.DOCX]

Supplementary Material

# Supplementary Figures


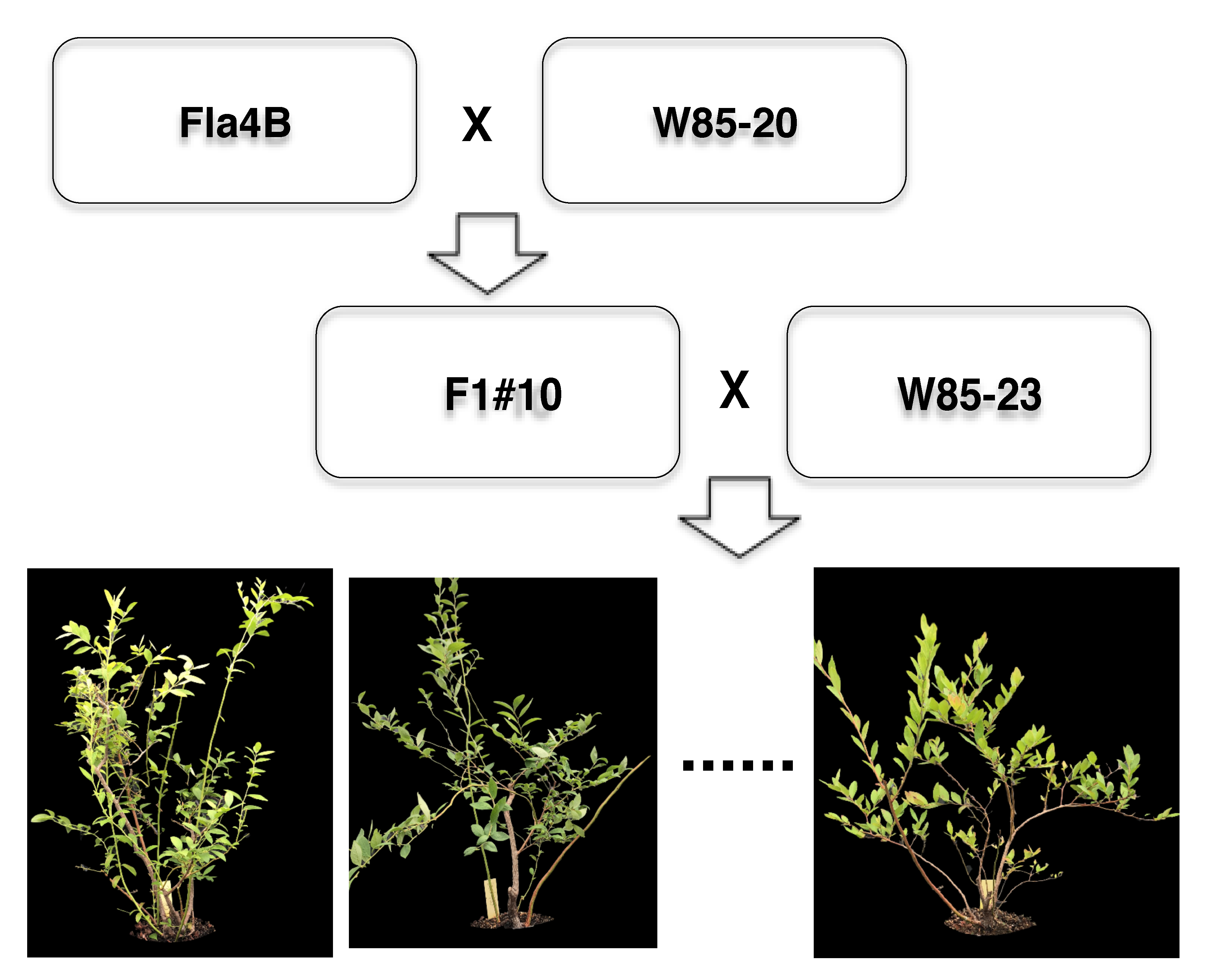


**Supplementary Figure 1.** Mapping population construction. Diploid blueberry pseudo-backcross interspecific population was created by crossing the parent F_1_#10 (*Vaccinium darrowii* Fla4B × *Vaccinium corymbosum* W85-20) with the parent W85-23 (*Vaccinium corymbosum*).

**Supplementary Figure 2.** SNP density along blueberry genome haplotype 1. Tracks from outside to inside: tetraploid blueberry genome chromosomes 1-12, SNP density and gene density calculated in 50 Kbp window sizes. SNP position was determined by aligning 150 bp flanking sequences back to the tetraploid blueberry genome haplotype 1.





**Supplementary Figure 3.** Synteny between previous genetic map based on mainly SSR markers (Schlautman et al., 2018) and current high density genetic map based on SNP marker bins.


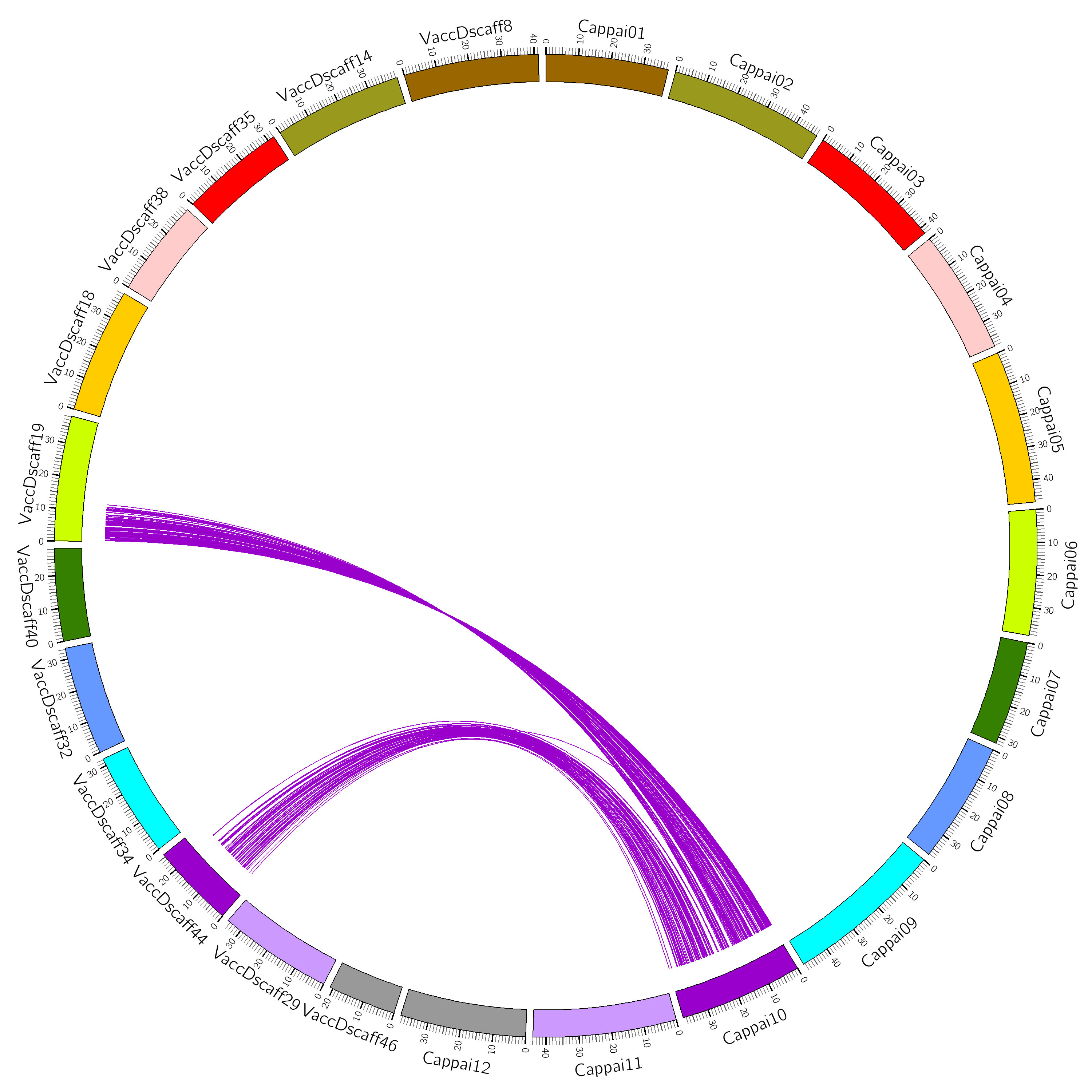


**Supplementary Figure 4.** Syntenic hits between LG10 of high-density tetraploid genetic map from the Munoz laboratory (Cappai et al., 2020) and tetraploid blueberry genome, haplotype 3. Syntenic hits to regions of VaccDScaff19 and VaccDScaff44 were found.

# Supplementary Tables

**Supplementary Table 1**

Attached Supplementary_Table1.xlsx file.

The file Supplementary Table 1 provides a summary of population re-sequencing and mapping statistics. This file contains 10 columns by 124 rows.

**Supplementary Table 2**

Attached Supplementary_Table2.xlsx file.

The file Supplementary Table 2 provides detailed information for markers comprising high density diploid blueberry genetic map. This includes SNP ID, SNP location, allele, linkage group (LG) location, Bin ID, genetic position (cM), 150 bp flanking sequence, and tetraploid genome position. The file contains 8 columns by 17,489 rows.

**Supplementary Table 3**

Attached Supplementary_Table3.xlsx file.

The file Supplementary Table 3 provides a summary of QTL results for all the traits analyzed. Traits include chilling requirement, cold hardiness, fruit quality-related traits, and traits related to timing of various developmental stages. How the population was phenotyped for chilling requirement and cold hardiness is described in detail in Rowland et al. (2014). How the population was phenotyped for the remaining traits is described in detail in Rowland et al. (2020). The file contains 11 columns by 40 rows.

**Supplementary Table 4**

Attached Supplementary_Table4.xlsx file.

The file Supplementary Table 4 provides a list of genes and their annotations from the vicinity of the chilling requirement QTL on LG05 and the cold hardiness QTL on LG10. The genes are from the diploid blueberry draft genome. The file contains 13 columns by 392 rows.

**Supplementary Table 5**

Attached Supplementary_Table5.xlsx file.

The file Supplementary Table 5 provides a list of genes and their annotations from the vicinity of the color QTL on LG02. The genes are from the diploid blueberry draft genome. The file contains 13 columns by 250 rows.

**Supplementary Table 6**

Attached Supplementary_Table6.xlsx file.

The file Supplementary Table 6 provides a list of genes and their annotations from the vicinity of the color QTL on LG10. The genes are from the diploid blueberry draft genome. The file contains 13 columns by 191 rows.

**Supplementary Table 7**

Attached Supplementary_Table7.xlsx file.

The file Supplementary Table 7 provides a list of genes and their annotations from the vicinity of the scar QTL on LG12. The genes are from the diploid blueberry draft genome. The file contains 13 columns by 132 rows.

**Supplementary Table 8**

Attached Supplementary_Table8.xlsx file.

The file Supplementary Table 8 provides a list of genes and their annotations from the vicinity of the 20SFirm QTL on LG06. The genes are from the diploid blueberry draft genome. The file contains 13 columns by 217 rows.
